# A mobile health app for real-time symptom monitoring in patients with cancer during COVID-19: feasibility, acceptability, and utility

**DOI:** 10.1186/s12885-026-15772-2

**Published:** 2026-02-20

**Authors:** Ruosi Shao, Jordan M. Neil, Meng Chen, Bethany Hannafon, Ryan Nipp, Audrey Montgomery, Summer G. Frank-Pearce, Katherine Moxley, Debra L. Richardson, Jacey Elliott, Lizbeth Benson, Amy Gin Gossett, Michael S. Businelle

**Affiliations:** 1 TSET Health Promotion Research Center, Stephenson Cancer Center, 655 Research Parkway, Suite 400, Oklahoma City, OK 73104 USA; 2 Department of Family and Preventive Medicine, University of Oklahoma Health Sciences, Oklahoma City, OK USA; 3 Department of Pediatrics, University of Oklahoma Health Sciences, Oklahoma City, OK USA; 4 Department of Obstetrics and Gynecology, Stephenson Cancer Center, University of Oklahoma Health Sciences, Oklahoma City, OK USA; 5 Department of Biostatistics and Epidemiology, Hudson College of Public Health, University of Oklahoma Health Sciences, Oklahoma City, OK USA; 6 Oklahoma Cancer Specialists and Research Institute, Tulsa, OK USA; 7 Institute for Social Research, University of Michigan, Ann Arbor, MI USA; 8 School of Communication, Florida State University, Tallahassee, FL USA; 9 Department of Psychology, Dana and David Dornsife College of Letters, Arts and Sciences, University of Southern California, Los Angeles, CA USA

**Keywords:** Cancer, Mobile health (mHealth), Patient-reported outcomes (PROs), Symptom monitoring, Oncology care

## Abstract

**Background:**

Patients undergoing cancer treatment often endure significant symptom burden, necessitating timely management to prevent severe complications. The COVID-19 pandemic highlighted the need for real-time remote symptom monitoring, particularly for cancer patients at heightened risk due to immunosuppression from cancer and chemotherapy.

**Methods:**

This 24-week, single-arm intervention study was conducted from July 2020 to May 2021 at the Stephenson Cancer Center Infusion Clinic in Oklahoma, USA. This study assessed the feasibility, acceptability, and utility of the Symptom Tracker smartphone app. The app prompted 128 patients receiving cytotoxic chemotherapy to report symptoms associated with cancer, cancer treatment, and COVID-19. Reports indicating elevated COVID-19 risk automatically triggered notifications to nursing staff for follow-up and testing. Primary outcomes included daily symptom reporting adherence and patient satisfaction.

**Results:**

Participants (*N* = 128) had a mean age of 57.6 years (SD = 13.0), with 77.3% being female. Approximately 10,000 daily symptom reports were completed, representing 46% of all study days. The app facilitated 302 patient-initiated contacts with cancer treatment staff. During the study period, seven participants (5.5%) tested positive for COVID-19 and were successfully triaged. Weekly feedback revealed that 76.8%-89.1% of participants considered the frequency of assessments “about right.”

**Conclusion:**

The Symptom Tracker app demonstrated feasibility, acceptability, and utility, as evidenced by high symptom reporting adherence, user satisfaction, and effective remote symptom management across a diverse patient population in Oklahoma. Our approach shows promise for supporting oncology care, enhancing real-time monitoring of symptom variability, and ensuring rapid response to critical symptoms.

**Trial Registration:**

ClinicalTrials.gov Identifier: NCT04397614, Registration date: May 22, 2020.

**Supplementary Information:**

The online version contains supplementary material available at 10.1186/s12885-026-15772-2.

## Background

Patients undergoing active cancer treatment often endure substantial symptom burden and treatment-related toxicities, which can rapidly escalate without timely intervention [1–4]. Studies have shown that high self-reported symptom severity (particularly for pain, appetite, shortness of breath, and tiredness) strongly predicts clinical deterioration, and deferred management can lead to recurrent emergency department visits, prolonged hospitalizations, readmissions, and severe declines in health-related quality of life [5–9]. In addition, clinicians often struggle to reliably detect patients’ symptoms during routine clinic visits due to patient underreporting and recall biases inherent in traditional retrospective assessments [10–13]. This highlights a crucial need for proactive, real-time symptom management to prevent escalation [14–18]. 

The integration of mobile health (mHealth) technology into oncology care has emerged as a promising solution for timely and effective symptom monitoring [19–21]. 91% of U.S. adults and 88% in rural areas own smartphones, enabling widespread adoption of remote monitoring solutions [22]. Smartphone-based ecological momentary assessments (EMAs)—brief, frequent, real-time surveys—capture moment-to-moment experiences [23, 24], reduce recall bias, and provide more accurate symptom measurements than traditional approaches that rely on patient recall over extended periods [25–27]. 

Empirical evidence suggests that early, proactive symptom monitoring can enhance patient-provider communication, improve symptom management, reduce emergency encounters, unplanned admissions and hospital stays, significantly improving quality of life and increasing survival for patients receiving cancer treatment [15, 28–33]. However, the efficacy of this EMA-based approach depends substantially on patient adherence, which varies depending on survey length, frequency, and perceived burden [34–36]. Research on cancer symptom monitoring has varied from daily to weekly assessments [21, 36–39]. For example, the Navigating Cancer ePRO (electronic patient-reported outcomes) platform required weekly self-reports of 14 common cancer-related symptoms via email or text, achieving a total of 16,299 weekly ePRO assessments provided by 1,841 participants, and resulting in a 64% survey completion rate over a 10-week period [38]. Results from this multisite community oncology trial reported 64% adherence on weekly ePRO assessments, however compliance declined from 72% to 52% over a 10-week period. Daily assessments, while offering greater accuracy to capture symptom variability and rapid changes, may also increase patient burden and lead to lower reporting adherence [36, 37]. A daily EMA study among patients with pediatric cancer reported mean adherence of 48.7%, declining from 77.1% in the first month, suggesting the difficulty of sustaining engagement with daily symptom reporting [40]. Therefore, the design of symptom monitoring interventions requires a careful balance between the need for detailed symptom reporting and the potential burden of it.

The COVID-19 pandemic further emphasized the need for real-time patient monitoring and remote interventions, particularly for immunocompromised populations [41–43]. Early detection and management of COVID-19 symptoms were crucial in reducing morbidity and mortality among this high-risk group, yet limited research has employed mHealth technology to assess, monitor, or manage COVID-19 symptoms in patients with cancer [44, 45]. The present study outlines the rapid development and implementation of the Symptom Tracker smartphone application (app) and evaluates its initial feasibility, acceptability, and utility among patients with cancer. Specifically, the study assessed patient adherence, acceptance, and engagement of daily prompted symptom reporting (primary aim) and explored predictors of completion rates based on patient characteristics, including cancer stage, rurality, and COVID-19 status (secondary aim). Findings inform the development of future remote symptom monitoring interventions that could be broadly incorporated into standard oncology care.

## Methods

### Study design and procedures

This study employed a 24-week, single-arm intervention conducted from July 2020 to May 2021 at the NCI-designated Stephenson Cancer Center (SCC) in Oklahoma, USA. The study protocol was approved by the Institutional Review Board at the University of Oklahoma Health Sciences (IRB12115) and was registered on ClinicalTrials.gov (NCT04397614). The Symptom Tracker app was developed, tested, and deployed within four weeks using the Insight™ mHealth platform [46] by the SCC mHealth Shared Resource.

Participants were recruited during visits to the SCC chemotherapy infusion clinic. Eligible patients underwent screening, provided informed consent, and downloaded the app on their personal or a study-provided smartphone. Participants then completed a baseline survey via the encrypted Insight™ platform. The app was programmed to prompt daily symptom reporting surveys 30 min after each participant’s preset wake time, with up to four additional reminders sent if the initial prompt was missed. No financial incentives or cost reductions were provided for study participation.

If participants reported symptoms indicative of COVID-19 (described in the *Measures* section), the app activated both automatic and patient-initiated alert mechanisms. Specifically, if two or more COVID-19 symptoms, or one symptom combined with recent COVID-19 exposure were reported, the app (1) automatically triggered an encrypted email alert to SCC nursing staff, including symptom details and the patient’s recent chemotherapy date, to facilitate immediate clinical follow-up for COVID-19 testing; and (2) sent an automated message prompting the participant to contact clinical staff via the in-app “Call Clinic Staff” button. Participants also had the option to proactively contact clinical staff using the same “Call Clinic Staff” button or submit additional symptom reports at any time using the “Report Symptoms” button, ensuring continuous access to clinical support beyond automated alerts and scheduled prompts.

**Fig. 1:**
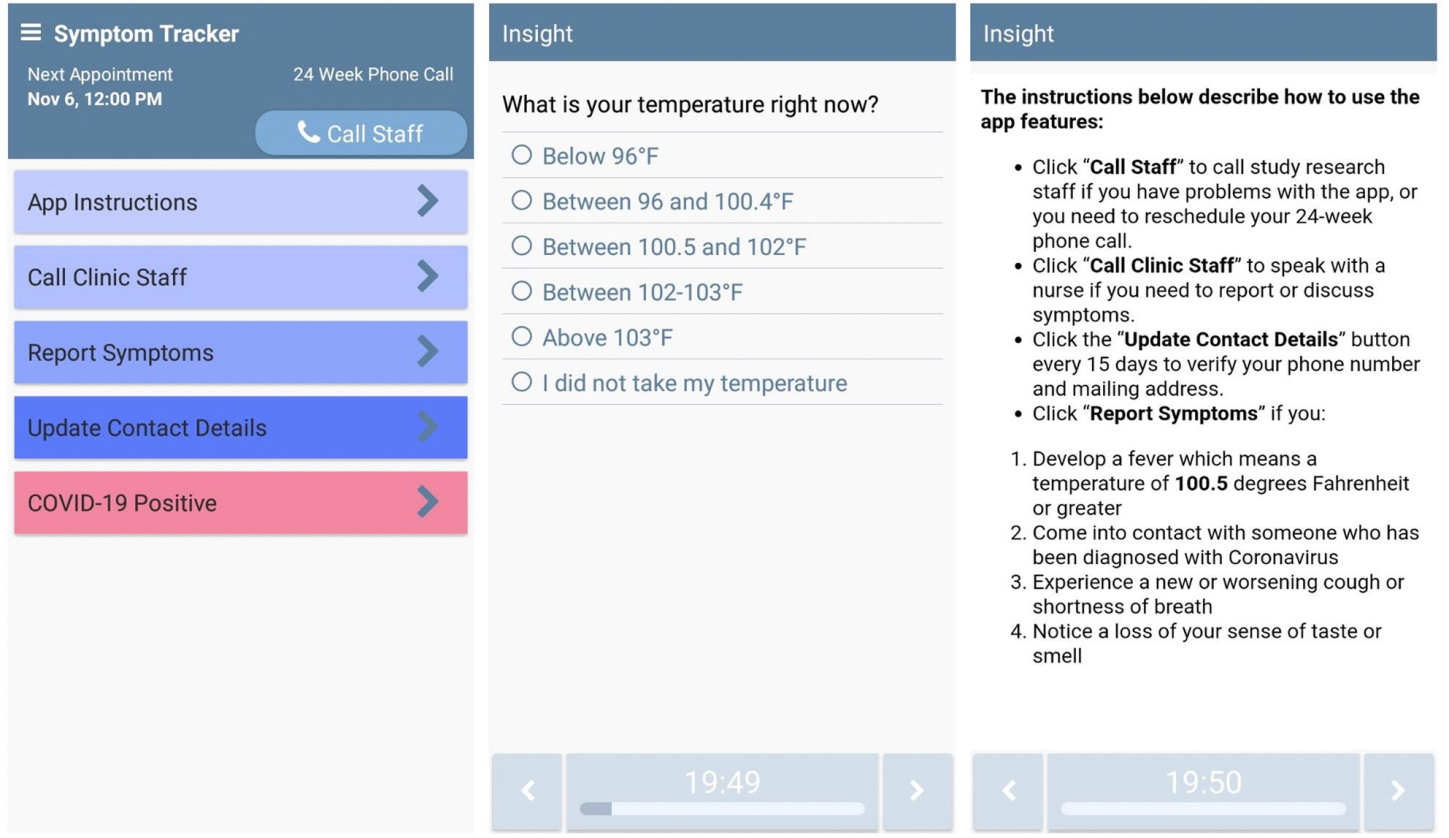
Screenshots of the symptom tracker app. Note: The app was designed to facilitate real-time symptom monitoring among patients undergoing cancer treatment during the COVID-19 pandemic. Left panel: home screen displaying key features including symptom reporting, contact options for staff, and COVID-19 status update. Center panel: example of a prompted symptom question (body temperature). Right panel: instructions detailing how and when to use app features, including automatic alerts for COVID-19 symptoms and patient-initiated reporting. These features supported continuous monitoring and rapid clinical follow-up, enhancing remote oncology care

**Fig. 2:**
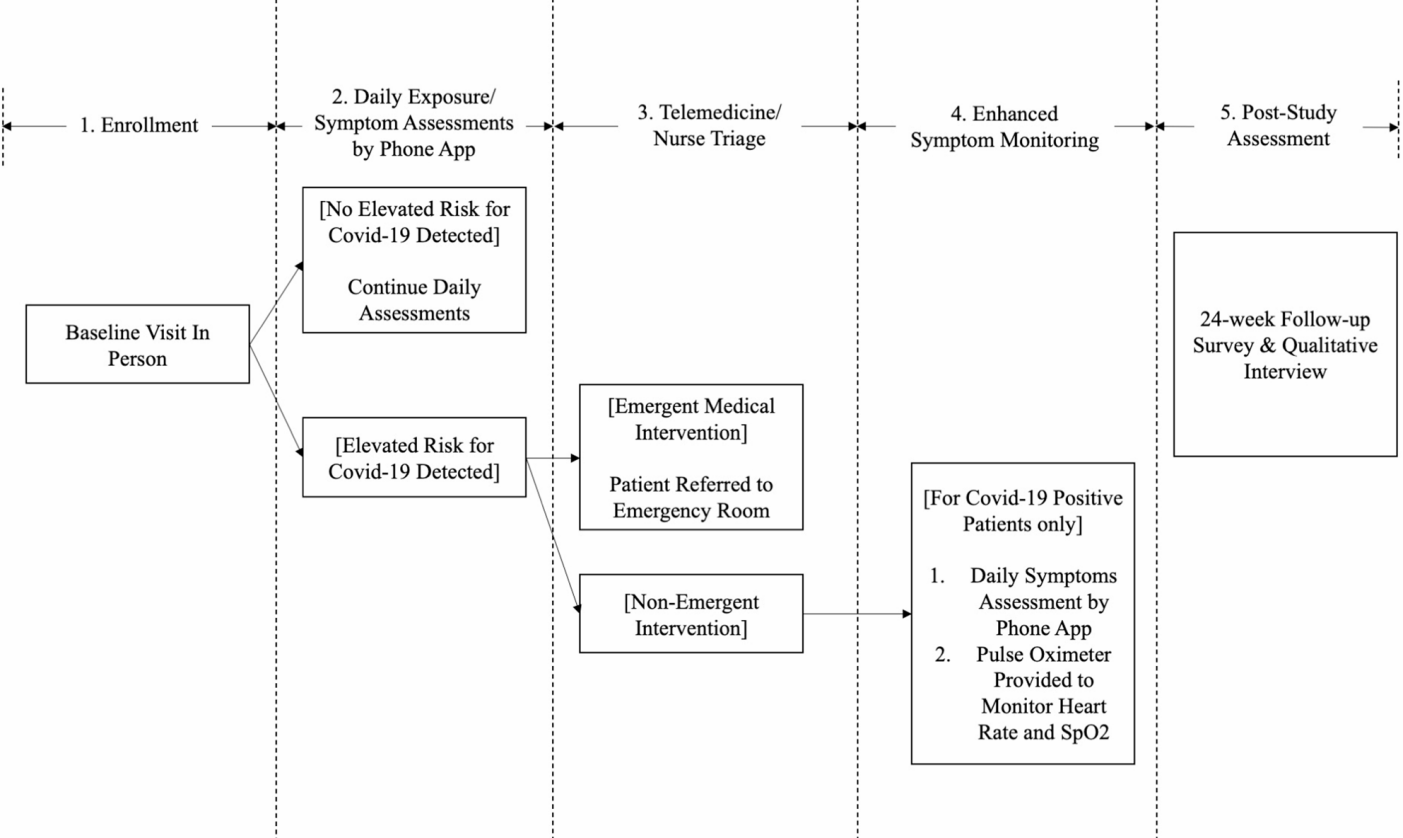
Participant flow for enhanced COVID-19 symptom monitoring. Note: This flowchart illustrates the sequential steps of the 24-week symptom monitoring protocol, beginning with in-person enrollment and daily symptom assessments via the Symptom Tracker app. If elevated COVID-19 risk was detected, the app triggered telemedicine triage by nursing staff. Patients requiring emergent intervention were referred to the emergency department, while those with non-emergent symptoms continued monitoring. COVID-19–positive patients entered an enhanced monitoring phase, receiving pulse oximeters and completing additional daily assessments for temperature, heart rate, and SpO₂ levels. All participants completed a 24-week follow-up survey and exit interview

Participants who screened positive for COVID-19 were instructed to select the “COVID-19 Positive” button in the app (see Fig. 1 for screenshots of the Symptom Tracker app), after which they received a pulse oximeter to monitor heart rate and blood oxygen levels, and entered an enhanced monitoring protocol (see Fig. 2 for participant flow). Following recovery and medical clearance, participants resumed standard symptom monitoring. At the conclusion of the 24-week study, participants completed a follow-up assessment through the app and participated in a semi-structured qualitative exit interview.

### Participants

Study inclusion criteria were: (1) English literacy at a 7th grade level or higher [47], (2) receiving cytotoxic chemotherapy or immunotherapy via intravenous infusion or oral medication at SCC, (3) at least 18 years of age, and (4) willing and able to complete surveys on a personal or loaned smartphone.

### Study measures

#### Sociodemographic and clinical characteristics

Sociodemographic characteristics, including age, sex, ethnicity, race, marital status, household income, education, health insurance, and ZIP code were collected at baseline. A binary rural-urban status variable was computed using ZIP-code-derived RUCA codes, with codes 1–3 classified as urban and codes 4–10 as rural [48]. Clinical characteristics, such as cancer type and stage, were retrieved from electronic health records (Table 1). Refer to the supplementary file for all the measures.

**Table 1:** Characteristics of enrolled participants and those completed the follow-up survey

Characteristics	Enrolled (*N* = 128)		Completed 24-Week Follow-Up (*N* = 61)	
	Mean/*N*	SD/%	Mean/*N*	SD/%
Age in years	57.6	13.0	58.0	13.3
Sex				
Female	99	77.3	50	82.0
Male	29	22.7	11	18.0
Ethnicity				
Hispanic	4	3.1	1	1.6
Non-Hispanic	124	96.9	60	98.4
Race				
White	99	77.3	47	77.0
Black or African American	9	7.0	6	9.8
Asian	1	0.8	—	—
American Indian/Alaska Native	11	8.6	4	6.6
More than one race	7	5.5	4	6.6
Other	1	0.8	—	—
Marital Status				
Single	15	11.7	7	11.5
Married or living with a significant other	74	57.8	35	57.4
Divorced	18	14.1	7	11.5
Widowed	14	10.9	7	11.5
Separated	7	5.5	5	8.2
Family Income (yearly)				
< 30,000	43	33.6	21	34.4
≥ 30,000	83	64.8	40	65.6
Refused to Answer	2	1.6	—	—
Education				
Kindergarten through 11th grade	9	7.0	3	4.9
High school or GED	24	18.8	11	18.0
Some college/technical school	47	36.7	24	39.3
Bachelor’s degree (Four-year college)	26	20.3	13	21.3
Post-graduate school	22	17.2	10	16.4
Health Insurance^a^				
Medicare	51	39.8	23	37.7
Medicaid	17	13.3	9	14.8
Military insurance	7	5.5	3	4.9
Job or personal insurance	62	48.4	31	50.8
Uninsured	9	7.0	4	6.6
Rural^b^				
Urban	80	62.5	39	63.9
Rural	48	37.5	22	36.1
Cancer Stage				
I	12	9.4	8	13.1
II	30	23.4	11	18.0
III	35	27.3	16	26.2
IV	42	32.8	20	32.8
Missing	9	7.0	—	—
Received a Study Phone				
Yes	92	71.9	40	65.6
Cancer Type				
Gynecologic	49	38.3	27	44.3
Breast	29	22.7	13	21.3
Gastrointestinal	13	10.2	4	6.6
Head and neck	11	8.6	4	6.6
Lung	9	7.0	2	3.3
Hematological	9	7.0	5	8.2
Genitourinary	4	3.1	3	4.9
Melanoma	2	1.6	1	1.6
Central nervous system	1	0.8	1	1.6
Unknown primary type	1	0.8	1	1.6

^a^Multiple responses were allowed for insurance types

^b^ZIP-code derived RUCA was used to classify urban (code 1–3) and rural (code 4–10) areas

#### Patient-reported symptoms via EMA

The daily EMA consisted of 14 questions: seven assessing COVID-19 symptoms (e.g., fever above 100.4 °F, new or worsening cough, shortness of breath, loss of taste or smell, fatigue, loss of appetite, and COVID-19 exposure within two weeks) and seven additional questions covering general health and psychological well-being (e.g., current affect, previous night’s sleep quality, perceived social support, and pain levels on a 0–10 scale).

Participants enrolled in the enhanced monitoring protocol following a positive COVID-19 screen responded to nine additional daily questions assessing body temperature, heart rate, and blood oxygen levels. If responses indicated the potential need for urgent medical attention, the app issued an automated alert prompting participants to call 911 or present to closest emergency department. Simultaneously, an encrypted notification was sent to the SCC nursing team to initiate follow-up.

Weekly assessments on Mondays included additional symptoms commonly associated with cancer treatment, such as fatigue, nausea, vomiting, pain, neuropathy, diarrhea, constipation, skin rash, and mouth sores. Participants were also asked to evaluate the burden of symptom reporting: “Consider the number of surveys that are prompted by the smartphone application. Is the number of assessments too high, about right, or not enough?”

#### Qualitative exit interview

Upon completing the 24-week intervention, participants were invited to a semi-structured phone interview for approximately 15 min. Interviews were recorded, transcribed, and assessed for general perceptions of the app, feedback on specific features, and suggestions for future improvements.

### Statistical analyses

Feasibility was assessed by calculating the percentage of days on which participants completed daily EMAs, including both prompted and self-initiated reports. Acceptability was operationalized by examining participants’ feedback on the frequency of daily assessments, obtained through weekly and 24-week follow-up surveys. A secondary measure of acceptability was examined by future use intentions: “Would you be interested in using a similar smartphone app in the future if needed?” Utility was evaluated based on the usage of on-demand app features (e.g., contacting SCC nursing staff through the app), and the successful triage of COVID-19-positive participants. Associations between EMA completion rates and patient characteristics, such as cancer stage, rurality, and COVID-19 testing results, were explored using ANOVA/independent t-test^1^, chi-squared tests^2^ or non-parametric alternatives (Kruskal-Wallis/Mann-Whitney-Wilcoxon and Fisher’s exact test). Additionally, patient characteristics were examined in relation to acceptability.

Qualitative data from exit interviews were analyzed using a deductive rapid analytic approach [49, 50]. Data were organized into summary templates corresponding to the a priori domains of inquiry—feasibility, acceptability, and utility. Content from the summary templates was synthesized and displayed into identified sub-themes, and illustrative quotes were used to highlight key findings (Table 2).

**Table 2:** Feasibility, Acceptability, and Utility Results from Rapid Qualitative Analysis (*N* = 45)

Domains	Subthemes	Exemplary Quotations
Feasibility	Consistency vs. Monotony of Symptom Reporting	“There were all always the same questions, so I kind of knew what to expect. Nothing ever really seemed to differ much, I think.” P-7024, Female, Age 31, Stage 5 Placenta Cancer, Urban Resident, COVID-19 Negative. “Sometimes it might have seemed repetitive answering the exact same question every day. I guess it’s really a question of do you think you need to check on somebody’s symptoms every single day?” P-7114, Male, Age 69, Stage 1 Uveal Melanoma, Rural Resident, COVID-19 Negative.
	Barriers to Completion of Symptom Reporting	“Probably the hardest thing of any of it was just waiting if I had something else going on at that time when it was when the phone was going to go off… especially I didn’t think about it on Sundays at church when we picked 9:00 o’clock that just being available for the app” P-7008, Female, Age 45, Stage 4 Ovarian Cancer, Urban Resident, COVID-19 Positive.
	Facilitators of Compliance of Symptom Reporting	“I got used to the sound [of the EMA notification], so it’s kind of like my alarm clock. It would wake me up and I would do it right then and there” P-7030, Female, Age 63, Stage 1 Endometrial Cancer, Rural Resident, COVID-19 Negative.
Acceptability	Reassurance from being Asymptomatic	“The fact that I could say no to those Covid questions all the time allowed me to say hey, I don’t have Covid because I’m saying no to all these.” P-7031, Male, Age 66, Stage 1 Tonsillar Cancer, Rural Resident, COVID-19 Negative.
	Intrusiveness vs. Importance of Notification Prompts	“I think it’s intrusive. It’s like having an albatross around your neck.” P-7012, Female, Age 43, Stage 2 Cervical Cancer, Rural Resident, COVID-19 Positive. “I may not have been as consistent as I was if it wasn’t for the alarm. So even my family, if they heard the alarm go off in a different room, they knew what it was, then I’d be like short time to do your survey.” P-7127, Male, Age 69, Stage 4 Breast Cancer, Urban Resident, COVID-19 Negative.
Utility	Benefits of Remote Symptom Monitoring	“The fact that I needed a nurse or somebody reading over it… to make sure that everything is going OK. They were just a phone call away. By pushing a button… The nurse did have to call me and tell me I needed to go get checked again and be seen by a doctor because [of] my symptoms…Especially when [I’m] two hours away from the hospital.” P-7012, Female, Age 43, Stage 2 Cervical Cancer, Rural Resident, COVID-19 Positive.
	Inclusion of a Comment Box for Detailed Symptom Reporting	“Perhaps a comment section. For example, I ended up having a tonsillectomy in in the middle of all this. And when you know all of a sudden you’re having an increase in pain. I could then tell you.” P-7085, Female, Age 72, Neuroblastoma, No uniformly accepted staging, COVID-19 Negative.
	Provision of Graphical Summaries	“add a way to show graphically the responses from the last 30 days.” P-7067, Female, Age 29, Stage 5 Breast Cancer, Urban Resident, COVID-19 Negative.
	Inclusion of Social Support	“Give helpful hints…take the experiences of past patients and relate them to the patients that are going through it… tell people about they’re going to have diarrhea or go buy briefs.” P-7096, Female, Age 72, Stage 4 Colon Cancer, Urban Resident. COVID-19 Negative.

## Results

### Participant characteristics

The study enrolled 128 participants with a mean age of 57.6 years (range 25–84 years); 77.3% were female, 77.3% identified as White, and 57.8% were married. Most participants (93.8%) owned a smartphone, and 62.5% resided in urban areas. Over 30% of the cohort had a current diagnosis of stage IV cancer at baseline (Table 1). By the end of this study, seven participants were recorded as deceased.

### Feasibility

Of the 128 study participants, 121 (94.5%) completed at least one daily EMA, and 61 (47.7%) completed the 24-week follow-up survey. On average, participants completed EMAs on 46% of study days, resulting in a mean of 77.6 days per participant (SD = 62.1). In total, participants completed approximately 10,000 daily surveys without financial compensation. Results from a Kruskal-Wallis rank sum test indicated that female participants completed more EMAs than male participants (*χ*^2^ [1] = 3.95, *p* = 0.047). Among participants who completed at least one daily EMA, cancer stage was negatively associated with EMA completion rate, such that participants with a higher stage diagnosis completed fewer EMAs (F_(1,111)_ = 4.62, *p* = 0.034). No other sociodemographic or clinical characteristics were statistically significantly associated with EMA compliance or follow-up survey completion.

### Acceptability

Participants completed a total of 1,239 weekly surveys, with 87% indicating that the frequency of daily surveys was “about right.” Similar results were observed at the 24-week follow-up, where 85.2% of participants continued to find the daily survey frequency appropriate, and 83.6% expressed interest in using a similar app in the future. There were no significant differences in acceptability based on cancer stage, rurality, or COVID-19 testing results.

### Utility

**Fig. 3:**
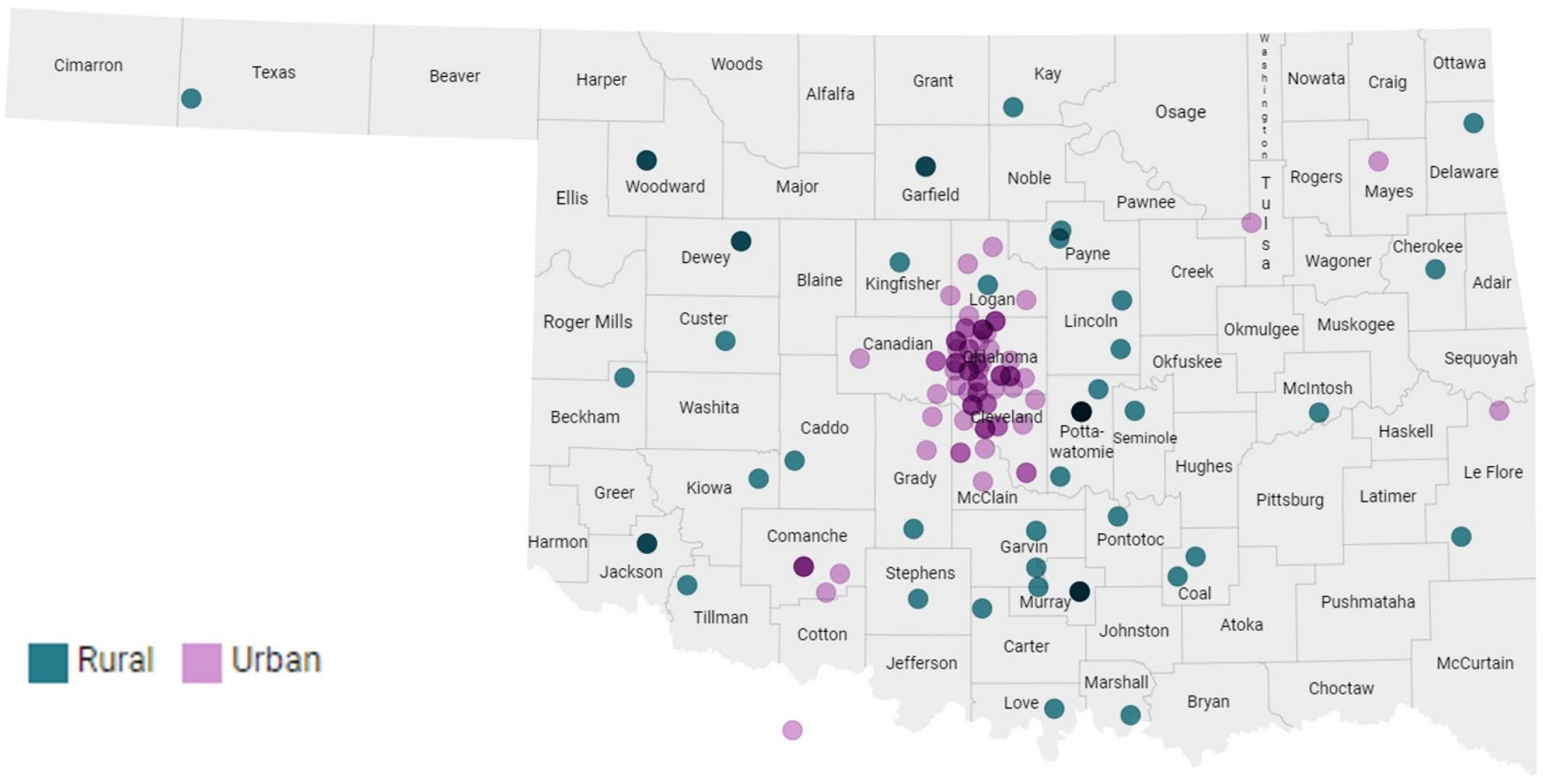
Participants’ home address zip codes by rural/urban residence. Note: Geographic distribution of study participants by ZIP code across Oklahoma, illustrating broad enrollment coverage spanning both rural and urban areas. Participants were recruited statewide, with concentrated enrollment near the Oklahoma City metro area and additional representation from rural counties throughout western, eastern, and southern Oklahoma. This distribution reflects successful engagement of both rural and urban populations in the symptom monitoring study

Throughout the study, 78.1% of participants accessed the “App Instructions” button at least one time (441 total uses), 66.4% clicked the “Report Symptoms” button (1,434 total uses), and 61.7% contacted the SCC clinic staff via the “Call Staff” button for a total of 302 times. Participants across Oklahoma (Fig. 3) completed over 10,000 symptom reports spanning 24 study weeks. During these EMAs, the reported symptoms included a body temperature above 100.4 °F on 12 days, a new or worsening cough on 216 days, shortness of breath on 66 days, loss of taste or smell on 96 days, exposure to COVID-19-positive individuals on 142 days, new fatigue on 560 days, and new loss of appetite on 308 days. Additionally, pain ratings above 5 on a 0–10 point scale were reported on 11.4% of the days.

COVID-19 testing was reported 29 times during the study, with the app’s COVID-19 risk algorithm triggered 68 times. This led to clinically meaningful actions by the study nurses, including immediate follow-up calls, mailing of pulse oximeters, and verification of test results. No instances were identified in which clinically significant symptoms were missed, indicating that the combined use of automated alerts and patient-initiated reporting effectively supported comprehensive symptom detection and response. Seven participants (5.5%) tested positive for COVID-19, with infection durations ranging from 3 to 15 days, and two participants reported reinfections. Another two COVID-19 cases were documented in participants’ medical charts. Participants who tested positive for COVID-19 completed a total of 100 enhanced monitoring assessments, with temperature, heart rate, and blood oxygen levels reported in 92.0%, 90.0%, and 89.0% of these EMAs, respectively. In the more comprehensive weekly EMAs, participants reported various symptoms, including fatigue (299 reports), neuropathy (270 reports), pain (245 reports), nausea (169 reports), constipation (141 reports), diarrhea (126 reports), mouth sores (80 reports), skin rash (59 reports), vomiting (47 reports), and other symptoms (27 reports).

### Qualitative exit interviews

#### Feasibility 

Most participants found the daily EMAs easy to complete, primarily due to the consistent nature of symptom reporting, which was appreciated for its predictability. However, non-completion occurred due to monotony and repetitiveness of the daily EMA measures. Barriers to EMA completion included competing life priorities and fatigue associated with their diagnosis and treatment (e.g., being too exhausted to respond to the survey or oversleeping and missing the scheduled prompts). Facilitators of EMA completion included using notification reminders as an alarm clock or a prompt for taking medication. 

#### Acceptability

Participants valued the reassurance that came from reporting that they were asymptomatic, which contributed to a sense of security about not having COVID-19. However, critiques arose regarding the intrusiveness of notification prompts, with some participants suggesting a shift to only self-initiated symptom reporting. Conversely, other participants found the prompting system effective in ensuring survey completion, noting that the notification sounds became so familiar that family members would encourage participants to complete the EMA upon hearing them. 

#### Potential Utility 

App utility was primarily discussed in the context of remote symptom monitoring, particularly for participants residing far from healthcare facilities. The app also served as a vital social connection, described as “a contact to the outside world” for participants who felt unwell or isolated. Although participants did not report technical difficulties or challenges, they offered suggestions to improve the app’s utility, including incorporating features such as a comment box for providing detailed symptom descriptions during unexpected health events, graphical summaries of symptom data to track trends over time, and social support features that could provide practical advice and peer experiences. These enhancements could potentially increase user engagement and improve the overall effectiveness of the app.

## Discussion

The Symptom Tracker app demonstrated feasibility, acceptability, and utility for remote real-time symptom monitoring in oncology care. Participants completed symptom reports on approximately 10,000 study days, yielding an overall adherence rate of 46%, comparable to the 48.7% adherence observed in a prior study of patients with pediatric cancer engaged in daily symptom reporting without incentives [40]. This level of adherence is clinically meaningful, as it was sufficient to detect and respond to potential COVID-19 symptoms in a timely manner. Notably, participants received no financial incentives to complete any study surveys (baseline, follow-up, or daily ecological momentary assessments), and engagement was sustained even as the urgency surrounding COVID-19 monitoring declined with the broader availability of testing and vaccines during the study period. The widespread use of the app among both rural (40%) and urban patients across Oklahoma indicates its potential to mitigate geographic barriers to care by supporting remote monitoring, timely interventions, and reducing healthcare access disparities. Participants expressed a high level of satisfaction with the frequency of daily prompts, with 87% considering the frequency “about right,” highlighting a successful balance between capturing detailed symptom data and minimizing patient burden. The frequent use of app’s features, such as contacting clinical staff, underscores its practical utility and real-time responsiveness. The symptom monitoring algorithm effectively identified patients requiring prompt clinical attention on 68 occasions, facilitating timely care interventions from the study nurse and enhancing patient safety. Seven participants tested positive for COVID-19 during the study, leading to more intensive symptom monitoring. Additionally, the app facilitated the tracking of various cancer-related symptoms and potential treatment side effects, suggesting that future studies could leverage such data for more targeted interventions.

### Strengths and limitations

This study had several strengths. First, it collected approximately 10,000 daily symptom reports from patients undergoing active cancer treatment, providing a granular understanding of symptom variability during treatment. In contrast, many previous studies have relied solely on weekly assessments [31] or collected daily assessments only among hospitalized patients [21]. Second, the 24-week study length offered an extended period of observation compared to other feasibility studies, allowing for a more comprehensive evaluation of the app’s performance [51, 52]. Third, nearly 40% of participants resided in rural areas, reflecting the catchment area of the SCC, which covers the entire state of Oklahoma. The cancer mortality rate in rural areas is higher than urban areas (189.9 vs. 173.2 per 100,000), with more pronounced disparities in specific cancer types (e.g., lung cancer) [53]. Therefore, demonstrating that the app was equally feasible, acceptable, and usable across both rural and urban settings was an important finding, indicating its potential to reduce geographic disparities in symptom monitoring. Finally, the rapid development and integration of the Symptom Tracker app into clinical workflows during the COVID-19 pandemic showcased the adaptability of the Insight™ platform and the scalability of smartphone apps for supporting remote symptom monitoring.

Study limitations include the single-arm pilot study design and single-site data collection, which limited the ability to compare outcomes with standard care. Additionally, the participant sample was heterogenous in terms of cancer type and stage, with individuals enrolled at various points in their treatment. This approach was chosen to increase successful accrual during COVID-19 when clinical staff was focused on providing care rather than recruiting participants for clinical trials. Finally, the study had a higher-than-expected attrition rate at the 24-week follow-up. Compensating participants for completing assessments may have increased EMA and follow-up completion rates [54, 55]. 

## Conclusion

The Symptom Tracker app demonstrated substantial feasibility, acceptability, and utility for remotely monitoring symptoms among cancer patients. mHealth-based symptom monitoring represents a promising approach to improve patient outcomes by facilitating real-time data capture, reducing recall bias, and enabling timely interventions. Future implementations should leverage adaptive EMA approaches to maximize adherence and integrate real-time management strategies to address patient symptoms effectively, improving care quality and patient experiences in oncology.

## Supplementary Information

Supplementary Material 1.

## Data Availability

The datasets generated and analyzed during the current study are not publicly available due to restrictions on sharing medical records but are available from the corresponding author on reasonable request.

## Footnotes

1 Or the Kruskal-Willis/Mann-Whitney-Wilcoxon test if the distributions violated the normal assumption.

2 Or Fisher’s exact tests if the frequency counts of any group in the comparisons were less than 5.
