# Supplementary material for: A mobile health app for real-time symptom monitoring in patients with cancer during COVID-19: feasibility, acceptability, and utility
Source: BMC Cancer. 2026 Feb 20;26:408. doi: 10.1186/s12885-026-15772-2 (PMC13032350; doi:10.1186/s12885-026-15772-2)
Supplement: Supplementary file 1 — Supplementary Material 1. [file 12885_2026_15772_MOESM1_ESM.docx]

**Supplement**

A. Measurement Table

A.1 Baseline Assessment

| **Variable** | **Measure** | **Scale** |
| --- | --- | --- |
| Age | What is your age? |  |
| Sex | What gender were you assigned at birth? | 0=Female, 1=Male |
| Ethnicity | Are you Hispanic or Latino? | 0=No, 1=Yes |
| Race | How would you best describe your race? | 1=White,  2=Black or African American,  3=Asian (Cambodia, China, India, Japan, Korea, Malaysia, Pakistan, Vietnam),  4=Native Hawaiian or Other Pacific Islander (Guam, Samoa),  5=American Indian / Alaska Native,  6=More than one race,  7=Other. |
| Marital Status | What is your present marital status? | 0=Single  1=Married or Living with significant other  2=Divorced  3=Widowed  4=Separated |
| Income | Which of these categories best describes your total combined family income for the past 12 months? This should include income (before taxes) from all sources, wages, rent from properties, social security, disability and/or veteran's benefits, unemployment benefits, workman's compensation, help from relatives (including child payments and alimony), and so on. | Dropdown type  0=Less than $9999,  1=$10000 to $19,999,  2=$20000 to $29,999,  3=$30000 to $39,999,  4=$40000 to $49,999,  5=$50000 to $59,999,  6=$60000 to $69,999,  7=$70000 to $79,999,  8=$80,000 to $89,999,  9=$90,000-$99,999,  10=$100,000 or more,  999=Refuse to Answer. |
| Education | How many years of education have you completed? | 0=Kindergarten through 11th grade,  1=High school or GED,  2=Some college/technical school,  3=Bachelor Degree (Four-Year College),  4=Post-graduate School (e.g., Master’s degree, M.D., Ph.D., DDS, Dr.P.H., etc.). |
| Insurance | Which type(s) of health insurance do you have? Please check all that apply. | 1=Medicare,  2=Medicaid/Soonercare,  3=Military Insurance,  4=Insurance from a job or private insurance,  5=I do not have health insurance |

A.2 Symptom Tracking

A.2.1 Daily Symptom Tracking – Pre-COVID-19 Exposure

| **Variable** | **Measure** | **Scale** |
| --- | --- | --- |
| Sleep | How would you rate the quality of your sleep last night? | 1=Very poor, 5=Very good |
| Support | Overall, how much social support did you receive from family, friends, and others yesterday? | 1=No support, 5=Total support |
| Medication | Did you take medication yesterday for any of the following? (check all that apply) | 0= None of the above,  1= Other,  2= Post Traumatic Stress Disorder,  3= anxiety disorder,  4= bipolar disorder,  5= Schizophrenia or schizoaffective disorder,  6= Depression. |
| Content | I feel content. | 1=Strongly disagree, 5=Strongly agree |
| Sad | I feel sad. | 1=Strongly disagree, 5=Strongly agree |
| Stressed | I feel stressed. | 1=Strongly disagree, 5=Strongly agree |
| Pain | Rate your current level of pain. | 0=None, 10=Worst possible |
| Covid1 | Do you have a temperature higher than 100.4°F? | 0=No, 1=Yes |
| Covid2 | Do you have a new or worsening cough, or a persistent cough? | 0=No, 1=Yes |
| Covid3 | Are you experiencing new or worsening shortness of breath? | 0=No, 1=Yes |
| Covid4 | Are you experiencing new loss of taste or sense of smell? | 0=No, 1=Yes |
| Covid5 | Have you been exposed to anyone who has been diagnosed with COVID- 19 in the last 2 weeks? | 0=No, 1=Yes |
| Covid6 | Are you experiencing fatigue? | 0=No, 1=Yes |
| Covid7 | Are you experiencing loss of appetite? | 0=No, 1=Yes |

A.2.1 Daily Symptom Tracking – Post-COVID-19 Exposure

| **Variable** | **Measure** | **Scale** |
| --- | --- | --- |
| Sleep | How would you rate the quality of your sleep last night? | 1=Very poor, 5=Very good |
| Support | Overall, how much social support did you receive from family, friends, and others yesterday? | 1=No support, 5=Total support |
| Medication | Did you take medication yesterday for any of the following? (check all that apply) | 0= None of the above,  1= Other,  2= Post Traumatic Stress Disorder,  3= anxiety disorder,  4= bipolar disorder,  5= Schizophrenia or schizoaffective disorder,  6= Depression. |
| Content | I feel content. | 1-Strongly disagree, 5-Strongly agree |
| Sad | I feel sad. | 1-Strongly disagree, 5-Strongly agree |
| Stressed | I feel stressed. | 1-Strongly disagree, 5-Strongly agree |
| Pain | Rate your current level of pain. | 0-None, 10-Worst possible |
| Vitals1 | What is your temperature this morning? | 0=I did not take my temperature this morning,  1=Below 96°F,  2=Between 96 and 100.4°F,  3=Between 100.5 and 102°F,  4=Between 102-103°F,  5=Above 103°F. |
| Vitals2 | What is your heart rate this morning? | 0=I have not checked my heart rate this morning  1=Below 60,  2=Between 60-100,  3=Between 100-120,  4=Over 120. |
| Vitals3 | What is your oxygen level this morning? | 0=I have not checked my blood oxygen level this morning,  1=Below 85%,  2=Between 85 and 92%,  3=Above 93%. |
| Covid9 | Is your fever responding to medicine? | 0=No,  1=Yes,  2=No longer have a fever. |
| Covid10 | Are you experiencing shortness of breath at rest or with minimal activity, such as dressing, eating, bathing? | 0=No, 1=Yes |
| Covid11 | Are you experiencing shaking chills? | 0=No, 1=Yes |
| Covid12 | Are you experiencing loss of your sense of taste or smell? | 0=No, 1=Yes |
| Covid13 | Are you experiencing any chest pain, slurred speech? | 0=No, 1=Yes |
| Covid14 | Have you developed new pain accompanied by color change or “coolness” in an extremity (arm, hand, leg, foot)? | 0=No, 1=Yes |

A.2.3 Weekly Symptom Tracking on Monday for Both Pre- and Post-COVID-19 Exposure

| **Variable** | **Measure** | **Scale** |
| --- | --- | --- |
| Self-rated Health | Which of the following apply to you? (check all that apply) (1=I smoke cigarettes, 2=I drink alcohol, 3=I don’t eat enough fruit and vegetables, 4=I weigh too much, 5=I do not get enough physical activity, 6=I do not get enough sleep, 7=None of these apply to me) | 1=I smoke cigarettes,  2=I drink alcohol,  3=I don’t eat enough fruit and vegetables,  4=I weigh too much,  5=I do not get enough physical activity,  6=I do not get enough sleep,  7=None of these apply to me. |
| Alcohol1 | In the past week, on how many days did you drink alcohol? | 0-7 |
| Alcohol2 | In the past week, on how many days did you have 5 or more standard drinks? | 0-7 |
| PHQ1 | Over the last week, how often have you been bothered by little interest or pleasure in doing things? | 0=Not at all,  1=Several days,  2=More than half the days,  3=Nearly every day. |
| PHQ2 | Over the last week, how often have you been bothered by feeling down, depressed, or hopeless | 0=Not at all,  1=Several days,  2=More than half the days,  3=Nearly every day. |
| GAD1 | Over the last week, how often have you been bothered by feeling nervous, anxious, or on edge? | 0=Not at all,  1=Several days,  2=More than half the days,  3=Nearly every day. |
| GAD2 | Over the last week, how often have you been bothered by not being able to stop or control worrying? | 0=Not at all,  1=Several days,  2=More than half the days,  3=Nearly every day. |
| Emotion | How difficult have emotions (such as feeling sad, down, nervous, or anxious) made it for you to do your work, take care of the things at home, or get along with other people? | 0 = not difficult at all,  1= somewhat difficult,  2 = very difficult,  3 = extremely difficult. |
| Chemo | In the past week, have you experienced an increase in any of the following symptoms? (Check all that apply) | 0=None of the below,  1=Fatigue,  2=Nausea,  3=Vomiting,  4=Pain,  5=Neuropathy,  6=Diarrhea,  7=Constipation,  8=Skin rash,  9=Mouth sores,  10=Other |
| Acceptability^*^ | Consider the number of surveys that are prompted by the smartphone application. Is the number of assessments ___. | 1=Too high,  2=About right,  3=Not enough. |

^*^ Acceptability was also measured in the follow-up survey to reflect overall attitude towards the symptom tracking app.

B. Participants’ Reported Acceptability of the Symptom Tracking App Across 24 Study Weeks.
